# Does the inclusion of societal costs change the economic evaluations recommendations? A systematic review for multiple sclerosis disease

**DOI:** 10.1007/s10198-022-01471-9

**Published:** 2022-05-20

**Authors:** B. Rodríguez-Sánchez, S. Daugbjerg, L. M. Peña-Longobardo, J. Oliva-Moreno, I. Aranda-Reneo, A. Cicchetti, J. López-Bastida

**Affiliations:** 1 Department of Applied Economics, Public Economics and Political Economy, University Complutense of Madrid, Pl. Menéndez Pelayo 4, 28040 Madrid, Spain; 2 Graduate School of Health Economics and Management (Alta Scuola Di Economia E Management Dei Sistemi Sanitari), Universitá Cattolica del Sacro Cuore, Rome, Italy; 3 Economic Analysis and Finance Department, Faculty of Law and Social Sciences, University of Castilla-La Mancha, 45071 Toledo, Spain; 4 Economic Analysis and Finance Department, Faculty of Social Sciences, University of Castilla-La Mancha, Avda. Real Fábrica de Seda s/n, 45600 Talavera de la Reina, Toledo Spain; 5 Faculty of Health Sciences, Universidad Castilla-La Mancha, 45600 Talavera de la Reina, Toledo Spain

**Keywords:** Economic evaluation, Health technology assessment, Informal care, Productivity losses, Multiple sclerosis, Societal perspective, Social costs, Cost-effectiveness, Cost-utility, D61, H0, I11, I15, I18

## Abstract

**Background:**

Multiple sclerosis imposes a heavy burden on the person who suffers from it and on the relatives, due to the caregiving load involved. The objective was to analyse whether the inclusion of social costs in economic evaluations of multiple sclerosis-related interventions changed results and/or conclusions.

**Methods:**

A systematic review was launched using Medline and the Cost-Effectiveness Analysis Registry of Tufts University (2000–2019). Included studies should: (1) be an original study published in a scientific journal, (2) be an economic evaluation of any multiple sclerosis-related intervention, (3) include productivity losses and/or informal care costs (social costs), (4) be written in English, (5) use quality-adjusted life years as outcome, and (6) separate the results according to the perspective applied.

**Results:**

Twenty-nine articles were selected, resulting in 67 economic evaluation estimations. Social costs were included in 47% of the studies. Productivity losses were assessed in 90% of the estimations (the human capital approach was the most frequently used method), whereas informal care costs were included in nearly two-thirds of the estimations (applying the opportunity and the replacement-cost methods equally). The inclusion of social costs modified the figures for incremental costs in 15 estimations, leading to a change in the conclusions in 10 estimations, 6 of them changing from not recommended from the healthcare perspective to implemented from the societal perspective. The inclusion of social costs also altered the results from cost-effective to dominant in five additional estimations.

**Conclusions:**

The inclusion of social costs affected the results/conclusions in multiple sclerosis-related interventions, helping to identify the most appropriate interventions for reducing its economic burden from a broader perspective.

## Introduction

A shift towards a societal perspective in the economic assessment of healthcare technologies, including not only the payer’s or the provider’s point of view (direct costs), but also the impact on patients and their families and the public/societal expenditure (indirect costs), has been observed over the last decade [1]. The inclusion of non-healthcare costs or social costs such as informal care and/or productivity loss is gaining more and more importance as advances in treatment options, innovation in health technologies and new methods of diagnosis have provided new models of care. Due to medical advances, the management of several diseases has shifted from acute diseases with mainly hospital-based treatment to chronic diseases relying more and more on outpatient care with support from informal caregivers. Considering a broad perspective is particularly important when, in addition to the healthcare resources used and the effects on patients’ health, it is intended to evaluate interventions which can generate other types of significant effects on other social dimensions such as labour productivity, non-professional care time (informal care) or the health and well-being of other agents as well as those of the patients [2–6].

Multiple sclerosis (MS) is an autoimmune and neurodegenerative condition that affects the brain and spinal cord, causing a disfunction in part of the nervous system’s ability to transmit signals due to damage to the insulating covers of the nerve cells [7]. Some of the most common symptoms of MS entail difficulty in walking, vision problems and problems with balance and co-ordination [8, 9]. Those symptoms might be present before the diagnosis of the disease, since 85% of the individuals who later develop the condition begin with an episode of neurological disturbance, which is commonly known as a clinically isolated syndrome, which might progress over days or weeks [10]. In patients of working age, these symptoms could lead to short and long periods of absence from work. Since those people with a first appearance of symptoms have not yet been diagnosed, the societal impact (due to loss of productivity) could have been underestimated.

MS is particularly interesting to study since it is a disease associated with considerable healthcare and other social costs, due to early onset of symptoms which commonly manifest themselves in childhood and early adulthood (20 s and 30 s), and with a debilitating pathogenesis, making the disease one of the most common causes of disability in younger adults. Even though most people with MS are diagnosed at 20 to 50 years old, a recent study has shown that individuals with a diagnosis of MS at younger ages (during childhood) may develop a more severe stage of the disease after longer periods of time, even as long as 32 years after the diagnosis, than those individuals with a later diagnosis, who might worsen after 18 years, and who also take longer to reach disability milestones [11]. The onset of the disease affects the ability to work, as a recent study has revealed that the proportion of patients below retirement age who are employed or self-employed decreases as the severity of the illness increases [12], imposing a great burden in terms of societal costs (informal care costs and costs due to sick leave and early retirement), which can reach 60% of the total lifetime costs [13]. Thus, the symptomatology of MS leads patients and their families to greater needs of care (outside the healthcare system) and a severe curtailment of working life. This debilitating state of health also results in a lower quality of life [13], a higher risk of death and shorter life expectancies than in the general population without the disease [14–18], even though the availability of new treatments might be reducing those differences. Furthermore, due to advances in treatment options and diagnostic criteria, the costs of the disease have shifted from being mainly those of hospitalisation, to being those of outpatient care, which now accounts for 80–90% of MS-related healthcare costs [19, 20].

Apart from studies which focus entirely on the estimation of healthcare costs, the literature in the field of cost-of-illness studies reveals that non-healthcare costs, mainly those resulting from the loss of work and the cost of care associated with the loss of patients’ autonomy due to disability, represent a very high cost for society, being even higher than healthcare costs [12]. Furthermore, as the disease progresses and affects patients’ health more severely, social costs increase progressively, both in absolute value and in proportion to the total cost of MS [21]. Thus, apart from the effects on patients, the health of the closest relatives is also affected, as well as other aspects directly related to their well-being [22, 23].

However, and although several non-medical costs related to MS have been identified [24], which are usually out-of-pocket expenses, the most substantial non-medical costs associated with the disease have been shown to be productivity losses and informal caregiving costs [12]. A recent European study in 16 countries has shown that among MS sufferers who are below retirement age, only about 50% are employed (range from 31 to 65%). Those with no disruption on the Expanded Disability Status Scale (EDSS) had a work capacity of 82%, but among those with maximum disability due to the disease, this capacity declined to 8% [12]. Studies have also shown that MS has a significant impact on family members; about 50% of MS sufferers receive informal care from family members, ranging from less than 50 h/month for people with mild symptoms to round-the-clock care for those with severe symptoms [12]. Moreover, as the disease becomes more debilitating over the years, work life and family life often become heavily affected, and there is a high impact on the health-related quality of life of the individual sufferers and their families, and a high impact on society as well [12, 25].

Despite the efforts developed in the field of burden and cost-of-illness studies, to the best of our knowledge, there is no evidence on the influence of considering the perspective of the society in economic evaluations of healthcare interventions on MS. The aim of the present work was, therefore, to study the effects of considering a broader perspective (societal) instead of the healthcare funder’s perspective in the economic assessment of MS-related healthcare interventions. Therefore, we tried to discover whether a consideration of society’s perspective would significantly modify the results and the recommendations of the economic evaluations performed.

## Methods

### Study design: search strategy and inclusion criteria

We performed a systematic literature review, using the methodological framework outlined in the Preferred Reporting Items for Systematic Reviews and Meta-Analyses (PRISMA) statement [26]. A search in the MEDLINE database using PubMed and the Cost-Effectiveness Analysis (CEA) Registry of Tufts University was performed, covering the period from 1st January 2000 to 31st October 2019. The two databases were selected to perform a comprehensive search covering a broad range of disciplines related to economic evaluations within the medical field. Since the CEA Registry is based on MEDLINE searches for articles using the keywords “QALYs”, “quality-adjusted” and “cost-utility analysis”, results would be expected to overlap [27]. However, studies have shown that searching in both databases ensures a more accurate search [28]. The search launched in PubMed included the subject headings (Mesh terms in PubMed) and targeted “keywords search” for the following two groups: (1) Economic evaluation (including the terms: “Costs and Cost Analysis”; “cost–effectiveness”; “cost–utility”; “cost–benefit”; “economic evaluation”; “economic analysis”; “QALY”; “quality-adjusted life years”) and (2) multiple sclerosis (including the terms: “multiple sclerosis”; “sclerosis”). We restricted our review to any original studies published in a scientific journal and including an economic evaluation of any intervention related to multiple sclerosis. However, studies were only included if social costs (informal care costs and/or productivity losses) were included in the analysis and results were provided separately for each perspective applied (healthcare provider/payer and societal perspectives). Moreover, studies had to use quality-adjusted life years (QALYs) as one of the outcomes of the analysis, and the results had to be given separately (or could be extracted) if other diseases were also included in the analysis. The search was restricted to studies conducted on humans and published in English. Studies were also excluded if they were reviews of economic evaluations, methodological studies or were not full economic evaluations, such as a cost-of-illness study, a burden-of-disease study or a budget impact analysis.

### Overview and definitions

This review focuses on the cost-utility analysis, which measures the health outcomes in QALYs and the costs of multiple sclerosis-related interventions. Therefore, to reflect the true range of costs and outcomes of multiple sclerosis interventions, the analysis examines the interventions from both the healthcare perspective and the societal perspective.

For the purpose of the study, the concept of healthcare costs has been defined in accordance with the OECD report of 2000 (revised in 2011) “System of Health Accounts methodology proposed by the Organisation for Economic Cooperation and Development (OECD)” [29]. It is important to note that according to that definition, professional long-term care would be part of direct healthcare costs and is described as follows: “Total long-term care consists of a range of medical/nursing care services, personal care services and assistance services that are consumed with the primary goal of alleviating pain and suffering or reducing or managing the deterioration in health status in patients with a degree of long-term dependency”. Hence, in this study, direct healthcare costs included all medical resources as well as professional caregiving, which would constitute the perspective of the healthcare provider/payer, whereas social costs referred mainly to informal care (provided by non-professional caregivers) and to productivity costs due to loss of productivity. Therefore, the societal perspective would then add to the healthcare provider/payer’s perspective the costs due to informal care and productivity losses.

### Study selection procedure and data extraction

To eliminate bias and errors in the methodology, the process of study selection and data extraction was double-blinded and was conducted using peer review. We followed a three-stage selection method based on the inclusion and exclusion criteria described above. First, BRS screened the lists of selected titles and abstracts identified in the electronic literature searches, and full texts were retrieved if the abstract indicated that the study was an economic evaluation of multiple sclerosis or sclerosis and if it was written in English. Second, based on the inclusion criteria, two investigators (SD and BRS) independently conducted a full-text review and assessed each paper as included, excluded, or unsure. The individual screening results were compared, and discrepancies were resolved by a third reviewer (LMPL or IAR) conducting a full-text review. Final agreement was achieved through discussion among the research team members to achieve consensus. Third, relevant data from the selected articles were extracted using an Excel-based data extraction table specifying the following information: author’s full name, year of publication, journal, title, intervention type, country and currency, discount rate (costs/outcomes), time horizon, perspective applied (healthcare provider/payer or societal), costs and QALYs as a consequence of the intervention against its comparator from both perspectives, the threshold assumed for the economic evaluation, costs included (healthcare and social costs), and the method used for calculating social costs. The data-extracting process was also double-blinded, using peer review with two independent researchers (BRS and SD) extracting the data. Disagreements in the data-extracting phase were settled by introducing a third researcher (LMPL or IAR).

### Data synthesis

Following the data extraction, a narrative synthesis of the results from the included studies was performed. To assess the influence of the inclusion of social costs (informal care costs and/or productivity losses) on the result and conclusion of the economic evaluations of total costs of MS interventions, information about the incremental cost-utility ratios (ICURs) was collected or calculated (if not given by the original authors of the study) from both perspectives. The ICURs were then compared to determine whether the inclusion of social costs in the analyses had affected the conclusion or results of the study based on the threshold value assumed by the authors. In this sense, two options could produce changes in results or in the conclusions. We recorded a change in the conclusions when the decision about the adoption of a new technology was changed as a result of the inclusion of the social costs. For instance, from the healthcare perspective, the ICUR was above the threshold value, so the assessed technology was not recommended. However, when social costs were included, the ICUR was below the corresponding threshold. On the other hand, a change in results was identified when social costs were introduced and (1) the ICUR fell below the threshold (as in the previous case referred to, when a change in results led to a change in conclusions) or (2) the intervention became cost-saving (although it was previously already cost-effective but had a positive ICUR). It is important to stress that a significant change in the results may not change the conclusions of the analysis, as explained in the latter case. For example, an intervention assessed with a favourable ICUR from the healthcare perspective would already be recommended. If the inclusion of social costs made the ratio significantly more favourable (or even dominant), there would have been a change in the results of the economic evaluation without leading to a change in the conclusions, as the evaluated intervention would have already been recommended from the healthcare perspective.

## Results

**Fig. 1:**
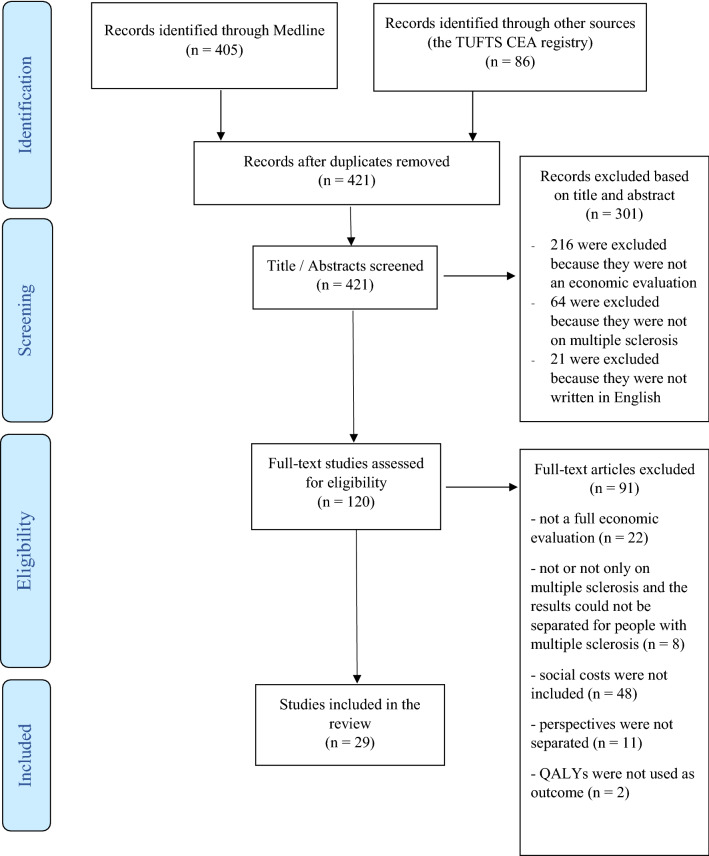
PRISMA flowchart of the search strategy

Based on the literature search in the 2 databases, 421 records were retrieved, after dropping duplicates (Fig. 1). After reviewing titles and abstracts, 301 were excluded because they did not meet the study criteria, leaving 120 records for full-text review. From these, 91 were additionally excluded for the following reasons: 22 were not full economic evaluations and eight were not evaluations of multiple sclerosis (or, if other diseases were included in the analysis, the results regarding MS could not be separated). Out of the 61 economic evaluations on multiple sclerosis that were excluded, 48 did not include social costs in their estimations, 11 did not separate perspectives (healthcare payer/provider from the societal perspective) and two papers did not use QALYs as outcome. Hence, 29 studies met the inclusion criteria and were finally included in the literature review [30–58].

### Study characteristics

Almost three-quarters of the studies considered either multiple sclerosis in general (11 studies, 38%), without specifying the type of MS [33, 36, 39, 42, 44, 47, 51–54, 57], or relapsing–remitting multiple sclerosis in another 11 studies [31, 34, 35, 37, 38, 40, 41, 43, 50, 55, 56]. Another two studies exclusively referred to people with secondary-progressive multiple sclerosis [45, 46]; one study focussed on slow-progression multiple sclerosis [30], progressive multiple sclerosis [32] and subsequent multiple sclerosis [49]; and another one on relapsing–remitting or secondary multiple sclerosis [48] and on secondary-progressive or progressive relapsing multiple sclerosis [58].

Of the 29 studies included in the analysis, 8 were performed in the United Kingdom [30, 32, 38–40, 51, 53, 57]; 7 in the United States [31, 35, 36, 50, 52, 54, 58]; 5 in Sweden [33, 44–47]; 2 were performed in France [34, 48]; 2 in Italy[37, 49]; 2 Iran [41, 56]; and 1 study was performed in Canada [42], 1 in Serbia [43] and 1 in Finland [55] (Table 1).

**Table 1:** Descriptive information on the selected studies (*n* = 29)

First author and publication year	Type of multiple sclerosis	Intervention type	Country and currency	Discount rate (costs/outcomes)	Time horizon	Perspective applied	Costs included	Method used for calculating social costs (items included)
Ball (2015) [30]	Slow progression multiple sclerosis	Non-pharmacological treatment (cannabinoids)	United Kingdom (2010/2011£)	3.5%; 3.5%	3 years	Healthcare provider’s perspective (societal perspective in a secondary analysis)	Healthcare costs: intervention and medication costs, hospital admissions, primary and acute care services, alternative practitioners, formal personal care services, home adaptations and equipment, day care, respite care, treatment-related travel. Social costs: informal care	Informal care: replacement-cost method
Bell (2007) [31]	Relapsing–remitting multiple sclerosis	Care delivery/pharmaceutical (disease-modifying therapy: injectable medication)	United States (2005$)	3%; 3%	Lifetime	Societal perspective (healthcare provider’s perspective could be extracted from the tables)	Healthcare costs: drug acquisition costs, health state-related costs. Social costs: productivity losses	Productivity losses: human capital approach
Bogosian (2015) [32]	Progressive multiple sclerosis	Non-pharmacological treatment	United Kingdom (2012/2013£)	n.a.; n.a	3 months	Societal perspective (healthcare provider’s perspective could be extracted from the text)	Healthcare costs: hospital (inpatient visits and hospitalizations) and community health and social care costs. Social costs: informal care	Informal care: n.a
Caloyeras (2012) [33]	Multiple sclerosis	Pharmaceutical (disease-modifying therapy: injectable medication)	Sweden (2009 SEKs)	3%; 3%	Lifetime	Societal perspective (healthcare perspective could be extracted from tables)	Healthcare costs: inpatient and ambulatory care, tests, medications, adverse event costs, services and investments. Social costs: informal care and productivity losses	Productivity losses: human capital approach (early retirement and absenteeism) Informal care: n.a
Chevalier (2016) [34]	Relapsing–remitting multiple sclerosis	Pharmaceutical (disease-modifying therapy: oral medication)	France (2013€)	4%; 4%	Lifetime	Societal perspective and healthcare payer’s perspective	Healthcare costs: treatment annual acquisition, administration and monitoring costs, inpatient care, ambulatory care, tests, prescription drugs other than DMTs, investments in additional resources for care, state costs and costs associated with relapses. Social costs: productivity losses	Productivity losses: human capital approach (early retirement, temporary disability and absenteeism)
Earnshaw (2009) [35]	Relapsing–remitting multiple sclerosis	Pharmaceutical (disease-modifying therapy: injectable + infused medications)	United States (2007$)	3%; 3%	Lifetime	Societal perspective and healthcare payer perspective	Healthcare costs: medical costs, non-medical costs (devices and investments to adapt living conditions).Social costs: productivity losses	Productivity losses: human capital approach (absenteeism)
Frasco (2017) [36]	Multiple sclerosis	Care delivery/pharmaceutical (disease-modifying therapy: infused medication)	United States (2016$)	3%; 3%	30 years	Societal perspective (healthcare provider’s perspective could be extracted from the tables)	Healthcare costs: inpatient and outpatient admissions, office visits to physicians and other healthcare professionals, examinations, medications (non-DMT prescription drugs, over-the-counter medicines), medical devices, and alterations to the house and services. Social costs: productivity losses and informal care	Productivity losses: human capital approach (early retirement, temporary disability and absenteeism) Informal care: opportunity-cost method (paid and unpaid time)
Furneri (2019) [37]	Relapsing–remitting multiple sclerosis	Pharmaceutical (disease-modifying therapy: infused medication)	Italy (2015€)	3.5%; 3.5%	50 years	Societal perspective (healthcare provider’s perspective could be extracted from the tables)	Healthcare costs: cost of disability, treatment acquisition, administration, monitoring, relapses, adverse events-related costs Social costs: productivity losses	Productivity losses: human capital approach
Gani (2008) [38]	Relapsing–remitting multiple sclerosis	Pharmaceutical (disease-modifying therapy: infused medication)	United Kingdom (2006£)	3.5%; 3.5%	30 years	Societal perspective (healthcare provider’s perspective in the sensitivity analysis)	Healthcare costs: inpatient admissions, nursing home care, day admissions, rehabilitation, healthcare specialists, diagnostic tests, non-DMT drug costs, home visits by nurses. Social costs: productivity losses	Productivity losses: n.a
Gras (2016) [39]	Multiple sclerosis	Pharmaceutical (cannabis-derived spray)	United Kingdom (2014£)	3.5%; 3.5%	30 years	Healthcare provider’s perspective (societal perspective in a secondary analysis)	Healthcare costs: community-based visits, outpatient clinic visits, A&E visits, hospital admission, home care visits, equipment costs. Social costs: informal care	Informal care: n.a
Hettle (2018) [40]	Relapsing–remitting multiple sclerosis	Pharmaceutical (disease-modifying therapy: oral + infused medications)	United Kingdom (2016£)	3.5%; 3.5%	50 years	Healthcare provider’s perspective (societal perspective in a secondary analysis)	Healthcare costs: drug acquisition, drug administration, and drug monitoring. Social costs: informal care and productivity losses	Productivity losses: n.a. Informal care: n.a
Imani (2012) [41]	Relapsing–remitting multiple sclerosis	Pharmaceutical (disease-modifying therapy: injectable medication)	Iran (2011$)	7.2%, 7.2%	Lifetime	Societal perspective (healthcare provider’s perspective could be extracted from the tables)	Healthcare costs: drug acquisition and health state-related costs. Social costs: productivity losses	Productivity losses: human capital approach
Iskedjian (2005) [42]	Multiple sclerosis	Pharmaceutical (disease-modifying therapy: injectable medication)	Canada (2001 CAN$)	5%; 5%	15 years	Societal perspective and healthcare payer’s perspective	Healthcare costs: medications, pharmacist visits, medication administration fees, physician fees, diagnostic procedures, laboratory testing fees, hospitalisation costs. Social costs: productivity losses	Productivity losses: human capital approach (paid and unpaid time)
Jankovic (2009) [43]	Relapsing–remitting multiple sclerosis	Pharmaceutical (disease-modifying therapy: injectable medication)	Serbia (2008 RSDs)	3%; 3%	40 years	Societal perspective and healthcare payer’s perspective	Healthcare costs: drug acquisition costs and health state-related costs. Social costs: productivity losses	Productivity losses: human capital approach
Kobelt (2008) [44]	Multiple sclerosis	Pharmaceutical (disease-modifying therapy: infused medication)	Sweden (2005€)	3%; 3%	20 years	Societal perspective and healthcare payer’s perspective	Healthcare costs: MS-related inpatient and outpatient care, rehabilitation, tests and drugs, walking aids and wheelchairs as well as nurse visits, home help and personal assistants, transfer to their home or car. Social costs: productivity losses and informal care	Productivity losses: n.a. (absenteeism and early retirement) Informal care: n.a
Kobelt (2000) [46]	Secondary progressive multiple sclerosis	Pharmaceutical (disease-modifying therapy: injectable medication)	Sweden (1998$)	3%; 3%	10 years	Societal perspective (healthcare provider’s perspective in the sensitivity analysis)	Healthcare costs: hospitalisation, medical, and other visits (visits to physicians, nurses, and to rehabilitation centres as well as to paramedical practitioners), prescription and non-prescription drug usage, community and other services, adaptations of the home or the workplace, medical and other devices purchased. Social costs: productivity losses and informal care cost	Productivity losses: n.a. (absenteeism, temporary disability and early retirement) Informal care: n.a
Kobelt (2002) [47]	Secondary progressive multiple sclerosis	Pharmaceutical (disease-modifying therapy: injectable medication)	Sweden (2000$)	3%; 3%	10 years	Societal perspective (healthcare provider’s perspective could be extracted from the tables)	Healthcare costs: detection, treatment, rehabilitation, long-term care, and investments. Social costs: productivity losses and informal care	Productivity losses: n.a. (absenteeism, temporary disability and early retirement) Informal care: n.a
Kobelt (2009) [48]	Multiple sclerosis	Care delivery/medical procedure	France (2007€)	3%; 3%	10 and 20 years	Societal perspective (healthcare provider’s perspective could be extracted from the tables)	Healthcare costs: inpatient care, ambulatory care, tests, drugs, investments, home help services. Social costs: productivity losses and informal care	Productivity losses: human capital approach (absenteeism, temporary disability and early retirement) Informal care: n.a
Kobelt (2003) [45]	Relapsing–remitting or secondary multiple sclerosis	Pharmaceutical (disease-modifying therapy: injectable medication)	Sweden (1999€)	3%; 3%	10 years	Societal perspective and healthcare payer’s perspective	Healthcare costs: drug costs and health state- and relapse-related costs. Social costs: productivity losses and informal care costs	Productivity losses: n.a. (absenteeism, temporary disability and early retirement) Informal care: n.a
Lazzaro (2009) [49]	Subsequent multiple sclerosis	Pharmaceutical (disease-modifying therapy: injectable medication)	Italy (2006€)	3%; 3%	25 years	Societal perspective and healthcare payer’s perspective	Healthcare costs: DMD and other drugs; outpatient diagnostic procedures, consultations and laboratory tests; hospitalisation (inpatient and day-hospital); physical therapy; walking aids (such as canes, tripods, manual and electrical wheelchairs). Social costs: productivity losses and informal care	Productivity losses: human capital approach (absenteeism) Informal care: opportunity-cost method (paid and unpaid time)
Mauskopf (2016) [50]	Relapsing, remitting multiple sclerosis	Pharmaceutical (disease-modifying therapy: oral medication)	United States (2015$)	3%; 3%	20 years	Healthcare provider’s perspective (societal perspective in a secondary analysis)	Healthcare costs: acquisition, administration, and monitoring costs and relapse-related costs. Social costs: productivity losses and informal care	Productivity losses: n.a. Informal care: n.a
Mosweu (2017) [51]	Multiple sclerosis	Care delivery	United Kingdom (2009£)	n.a.; n.a	1 year	Societal perspective and healthcare payer’s perspective	Healthcare costs: medication, inpatient, outpatient appointments, laboratory tests and scans, emergency department, contact with community professionals.Social costs: productivity losses and informal care	Productivity losses: human capital approach (absenteeism) Informal care: replacement cost method
Noyes (2011) [52]	Multiple sclerosis	Pharmaceutical (disease-modifying therapy: injectable medication)	United States (2008$)	3%; 3%	10 years	Societal perspective (healthcare provider’s perspective in the sensitivity analysis)	Healthcare costs: number of hospital admissions, outpatient treatments, emergency room visits, office visits, mental health visits, home health provider visits, home personal care use, and blood tests and MRI procedures. Social costs: productivity losses and informal care	Productivity losses: human capital approach Informal care: opportunity-cost method
Nuijten (2002) [53]	Multiple sclerosis	Pharmaceutical (disease-modifying therapy: injectable medication)	United Kingdom (1998£)	6%; n.a	Lifetime	Societal perspective and healthcare payer’s perspective	Healthcare costs: relapse-related direct costs. Social costs: productivity losses and informal care	Productivity losses: friction-cost method (paid time—absenteeism—and unpaid time) Informal care: replacement cost method
Pan (2012) [54]	Multiple sclerosis	Pharmaceutical (disease-modifying therapy: injectable medication)	United States (2011$)	3%; 3%	Lifetime	Societal perspective (healthcare provider’s perspective could be extracted from the tables)	Healthcare costs: treatment costs, relapse-related direct costs, EDSS-specific direct disease management costs (inpatient care, ambulatory care, tests, over-the-counter drugs and services). Social costs: productivity losses and informal care	Productivity losses: n.a. (absenteeism, presenteeism, early retirement and premature mortality) Informal care: n.a. (unpaid time)
Soini (2017) [55]	Relapsing–remitting multiple sclerosis	Pharmaceutical (disease-modifying therapy: oral + injectable medications)	Finland (2014€)	3%; 3%	15 years	Healthcare provider’s perspective (societal perspective in a secondary analysis)	Healthcare costs: drug, administration and monitoring costs, and adverse-event-related costs. Social costs: productivity losses	Productivity losses: n.a
Taheri (2019) [56]	Relapsing–remitting multiple sclerosis	Pharmaceutical (disease-modifying therapy: infused medication)	Iran (2018$)	7.2%; 3%	20 years	Societal perspective and healthcare payer’s perspective	Healthcare costs: drug acquisition costs, EDSS state-related costs, hospitalisation, AEs, rehabilitation, transport, wheelchair, and investments to adapt house or car. Social costs: productivity losses	Productivity losses: human capital approach (productivity losses due to morbidity and mortality)
Tosh (2014) [57]	Multiple sclerosis	Health education or behaviour	United Kingdom (2011£)	n.a.; n.a	9 months	Healthcare provider’s perspective (societal perspective in a secondary analysis)	Healthcare costs: intervention costs (staff, equipment, overheads) and NHS costs (GP appointment, neurology outpatient visit, NHS community health visit, social care visit, neurology inpatient visit, hospitalisation, accident and emergency visit) Social costs: productivity losses	Productivity losses: human capital approach (absenteeism)
Touchette (2003) [58]	Secondary progressive or progressive relapsing multiple sclerosis	Pharmaceutical (disease-modifying therapy: injectable + infused medications)	United States (2000$)	5%; 5%	10 years	Societal perspective and healthcare payer’s perspective	Healthcare costs: inpatient and outpatient visits. Social costs: productivity losses and informal care	Productivity losses: n.a. Informal care: n.a

Most often, the assessed interventions (22) were pharmaceutical treatments [33–35, 37–47, 49, 50, 52–56, 58]. However, three studies evaluated mixed interventions in care delivery and pharmaceutical procedures [31, 36, 48], two focussed on non-pharmacological treatments [30, 32], one was on a care delivery intervention [51] and another one was a health education or behaviour programme [57]. Among the pharmaceutical interventions, single or jointly with another type of intervention, only 1 concerned a symptom-disease management therapy, i.e. cannabis [39], and the remaining 23 evaluated a disease-modifying therapy: 12 studies assessed an injectable medication alone [31, 33, 41–43, 45–47, 49, 52–54], 5 additional articles individually evaluated an infused medication [36–38, 44, 56], and another two performed their economic evaluations solely on an oral medication [34, 50]. Two studies assessed the use of injectable and infused medications [35, 58], another one evaluated oral and infused medications together [40], and another article considered oral and injectable medications together [55].

Lifetime was the most frequently used time horizon for the evaluations [31, 33–35, 41, 53, 54] while the other studies used different time lapses depending on the type of intervention. With regard to the perspective applied, 12 studies used the societal perspective as the main point of view in the analysis [31–33, 36–38, 41, 46–48, 52, 54], in which the perspective of the healthcare payer/provider could be extracted from the text or tables, or appeared as a secondary analysis. Eleven studies performed the evaluation from both perspectives [34, 35, 42–45, 49, 51, 53, 56, 58], and the other 6 studies [30, 39, 40, 50, 55, 57] included the societal perspective in a secondary analysis (Table 1).

Only three studies exclusively included informal care costs [30, 32, 39], 11 studies only estimated labour productivity losses due to multiple sclerosis [31, 34, 35, 37, 38, 41–43, 55–57], while the other 15 studies included both types of social costs [33, 36, 40, 44–54, 58]. With regard to the valuation of informal care costs, if stated, the opportunity cost [36, 49, 52] and the replacement cost [30, 51, 53] methods were used equally in three studies. Another 12 studies did not provide information about the method used to value informal care costs. Two of the papers that applied the opportunity-cost method explicitly stated that both paid and unpaid time were valued [36, 49]. Frasco et al. (2017) [36] indicated that leisure time was valued as 65% of the average net income, whereas Lazzaro et al. (2009) [49] imputed a unit cost equal to €5.90 per hour for unpaid time. Pan et al. (2012) [54] only considered leisure time when evaluating informal care costs.

In the case of labour productivity losses, the human capital approach was used in 15 studies [31, 33–37, 41–43, 48, 49, 51, 52, 56, 57] if the authors explicitly reported the method used. Ten additional studies did not mention the approach used to value productivity losses. The friction-cost method was used in only one paper [53], differentiating the valuation of paid and unpaid time as follows: in the case of labour productivity losses due to absenteeism, each working hour lost was valued as 80% of the average value of a worker’s productivity; and the time lost by inactive individuals was valued at 40% of the average wage. Absenteeism was actually the main component of labour productivity losses, and was included in 14 studies [33–36, 44–49, 51, 53, 54, 57], whereas early retirement was included in 9 studies [33, 34, 36, 44–48, 54], temporary disability in 6 [34, 36, 45–48], premature mortality in 2 [54, 56] and presenteeism in 1 [54].

### Results of economic evaluations

Table 2 displays the results obtained from the 67 economic evaluations (EEs) resulting from the 29 articles included in this review.

**Table 2:** Incremental costs, QALYs and ICURs from the healthcare payer/provider’s and the societal perspectives

Estimation number	Healthcare payer/provider’s perspective				Societal perspective			Intervention to be implemented		Perspectives comparison		Threshold value
	Authors and publication year	∆Costs	∆QALYs	ICUR (cost/QALY)	∆Costs	∆QALYs	ICUR (cost/QALY)	Healthcare payer/provider’s perspective	Societal perspective	Do the results change? (yes/no)	Do the conclusions change? (yes/no)	
1	Ball et al. (2015) [30]	27,529	− 0.0001	− 275,290,000	30,294	− 0.0001	− 302,940,000	Comparator	Comparator	No	No	£20,000–30,000
2	Bell et al. (2007) [31]	58,741	0.222	264,599	57,174	0.222	258,465	Comparator	Comparator	No	No	$50,000
3	Bell et al. (2007) [31]	65,051	0.204	318,877	68,681	0.204	337,968	Comparator	Comparator	No	No	$50,000
4	Bell et al. (2007) [31]	79,097	0.198	399,480	82,410	0.198	461,301	Comparator	Comparator	No	No	$50,000
5	Bell et al. (2007) [31]	59,947	0.203	295,305	62,923	0.203	310,691	Comparator	Comparator	No	No	$50,000
6	Bogosian et al. (2015) [32]	− 720	0.01	− 72,000	− 2,285	0.01	− 228,500	Assessed intervention	Assessed intervention	No	No	£20,000
7	Caloyeras et al. (2012) [33]	− 167,000	0.479	− 348,643	− 344,000	0.479	− 718,163	Assessed intervention	Assessed intervention	No	No	n.a
8	Chevalier et al. (2016) [34]	− 7,902	− 0.281	28,121	− 3,684	− 0.281	13,110	Assessed intervention	Assessed intervention	No	No	€30,000–100,000
9	Chevalier et al. (2016) [34]	− 4,649	− 0.28	16,604	6	− 0.28	− 21	Assessed intervention	Comparator	Yes, when social costs are included, the incremental costs changed from negative to positive	Yes, the assessed intervention switched from cost-effective to dominated from the societal perspective and, hence, will not be adopted	€30,000–100,000
10	Chevalier et al. (2016) [34]	− 6,010	− 0.452	13,296	10,301	− 0.452	− 22,790	Assessed intervention	Comparator	Yes, when social costs are included, the incremental costs changed from negative to positive	Yes, the assessed intervention switched from cost-effective to dominated from the societal perspective and, hence, will not be adopted	€30,000–100,000
11	Chevalier et al. (2016) [34]	4,858	− 0.321	− 15,134	10,837	− 0.321	− 33,760	Comparator	Comparator	No	No	€30,000–100,000
12	Chevalier et al. (2016) [34]	− 3,692	− 0.224	16,482	849	− 0.224	− 3,790	Assessed intervention	Comparator	Yes, when social costs are included, the incremental costs changed from negative to positive	Yes, the assessed intervention switched from cost-effective to dominated from the societal perspective and, hence, will not be adopted	€30,000–100,000
13	Chevalier et al. (2016) [34]	45,103	− 0.25	− 180,412	49,460	− 0.25	− 197,840	Comparator	Comparator	No	No	€30,000–100,000
14	Earnshaw et al. (2009) [35]	66,564	0.134	496,746	− 56,322	0.134	− 420,313	Comparator	Assessed intervention	Yes, when social costs are included, the incremental costs changed from positive to negative	Yes, the assessed intervention switched from non-cost-effective to dominant and, hence, will be adopted	$50,000
15	Earnshaw et al. (2009) [35]	80,772	0.133	607,308	− 31,850	0.133	− 239,474	Comparator	Assessed intervention	Yes, when social costs are included, the incremental costs changed from positive to negative	Yes, the assessed intervention switched from non-cost-effective to dominant and, hence, will be adopted	$50,000
16	Frasco et al. (2017) [36]	− 255,498	0.84	− 304,164	− 287,713	0.84	− 342,515	Assessed intervention	Assessed intervention	No	No	$100,000
17	Furneri et al. (2019) [37]	62,850	1.52	41,349	− 18,928	1.52	− 12,453	Assessed intervention	Assessed intervention	Yes, the assessed intervention becomes dominant (not only cost-effective)	No	€50,000
18	Gani et al. (2008) [38]	35,530	1.9	18,700	4,300	1.9	2,263	Assessed intervention	Assessed intervention	No	No	£30,000
19	Gani et al. (2008) [38]	24,700	1.9	13,000	4,300	1.9	2,263	Assessed intervention	Assessed intervention	No	No	£30,000
20	Gras and Broughton (2016) [39]	3,836	0.35	10,891	− 33,609	0.35	− 95,423	Assessed intervention	Assessed intervention	Yes, the assessed intervention becomes dominant (not only cost-effective)	No	£30,000
21	Hettle et al. (2018) [40]	− 11,652	0.868	− 13,424	− 22,965	0.968	− 23,724	Assessed intervention	Assessed intervention	No	No	£30,000
22	Hettle et al. (2018) [40]	− 120,485	1.56	− 77,234	− 140,901	1.71	− 82,398	Assessed intervention	Assessed intervention	No	No	£30,000
23	Imani and Golestani (2012) [41]	125,101	0.204	613,240	123,909	0.204	607,397	Comparator	Comparator	No	No	$50,000
24	Imani and Golestani (2012) [41]	280,386	0.203	1,381,212	278,994	0.203	1,374,355	Comparator	Comparator	No	No	$50,000
25	Imani and Golestani (2012) [41]	2,323,420	0.198	11,734,444	230,970	0.198	1,166,515	Comparator	Comparator	No	No	$50,000
26	Imani and Golestani (2012) [41]	50,563	0.049	1,031,898	49,511	0.049	1,010,429	Comparator	Comparator	No	No	$50,000
27	Iskedjian et al. (2005) [42]	65,000	0.2856	227,586	55,000	0.2856	189,286	Comparator	Comparator	No	No	$50,000
28	Iskedjian et al. (2005) [42]	65,000	0.56	116,071	55,000	0.56	91,228	Comparator	Comparator	No	No	$50,000
29	Jankovic et al. (2009) [43]	6,560,000	0.6	10,933,333	6,800,000	0.6	11,333,333	Comparator	Comparator	No	No	RSD5,000,000
30	Jankovic et al. (2009) [43]	16,960,000	0.6	28,266,667	16,700,000	0.6	27,833,333	Comparator	Comparator	No	No	RSD5,000,000
31	Jankovic et al. (2009) [43]	16,960,000	0.6	28,266,667	16,600,000	0.6	27,666,667	Comparator	Comparator	No	No	RSD5,000,000
32	Jankovic et al. (2009) [43]	15,060,000	0.6	25,100,000	14,800,000	0.6	24,666,667	Comparator	Comparator	No	No	RSD5,000,000
33	Kobelt et al. (2008) [44]	13,010	0.34	38,145	− 3,830	0.34	− 11,265	Assessed intervention	Assessed intervention	Yes, the assessed intervention becomes dominant (not only cost-effective)	No	€50,000
34	Kobelt et al. (2000) [46]	102,800	0.162	634,600	55,500	0.162	342,700	Comparator	Comparator	No	No	$30,000; 40,000; 100,000
35	Kobelt et al. (2002) [47]	9,700	0.217	44,700	5,577	0.217	25,700	Comparator	Assessed intervention	Yes	Yes, the assessed intervention becomes cost-effective and will be adopted	$30,000
36	Kobelt et al. (2009) [48]	10,800	0.15	72,000	2,250	0.15	15,000	Comparator	Assessed intervention	Yes	Yes, the intervention becomes cost-effective and will be adopted	€30,000
37	Kobelt et al. (2009) [48]	11,000	0.13	84,615	2,000	0.13	15,385	Comparator	Assessed intervention	Yes	Yes, the intervention becomes cost-effective and will be adopted	€30,000
38	Kobelt et al. (2009) [48]	13,400	0.15	89,333	10,700	0.15	71,333	Comparator	Comparator	No	No	€30,000
39	Kobelt et al. (2009) [48]	11,000	0.13	84,615	8,700	0.13	66,923	Comparator	Comparator	No	No	€30,000
40	Kobelt et al. (2009) [48]	15,500	0.07	221,429	11,250	0.07	160,714	Comparator	Comparator	No	No	€30,000
41	Kobelt et al. (2009) [48]	13,000	0.05	260,000	9,250	0.05	185,000	Comparator	Comparator	No	No	€30,000
42	Kobelt et al. (2009) [48]	16,000	0.07	228,571	15,000	0.07	214,286	Comparator	Comparator	No	No	€30,000
43	Kobelt et al. (2009) [48]	13,250	0.05	265,000	12,250	0.05	245,000	Comparator	Comparator	No	No	€30,000
44	Kobelt et al. (2003) [45]	9,562	0.192	49,800	1,500	0.192	7,800	Assessed intervention	Assessed intervention	No	No	€50,000
45	Lazzaro et al. (2009) [49]	894	0.35	2,575	− 5,606	0.35	− 16,018	Assessed intervention	Assessed intervention	Yes, the assessed intervention becomes dominant (not only cost-effective)	No	€5,500
46	Mauskopf et al. (2016) [50]	− 33,059	0.359	− 92,086	− 39,896	0.359	− 111,131	Assessed intervention	Assessed intervention	No	No	n.a
47	Mauskopf et al. (2016) [50]	− 33,059	0.359	− 92,086	− 48,834	0.359	− 136,028	Assessed intervention	Assessed intervention	No	No	n.a
48	Mosweu et al. (2017) [51]	1,610	0.0053	303,774	2,871	0.0053	541,698	Comparator	Comparator	No	No	£60,000
49	Noyes et al. (2011) [52]	198,747	0.192	1,035,140	173,053	0.192	901,319	Comparator	Comparator	No	No	n.a
50	Noyes et al. (2011) [52]	218,535	0.173	1,263,207	194,307	0.173	1,123,162	Comparator	Comparator	No	No	n.a
51	Noyes et al. (2011) [52]	191,039	0.082	2,329,739	178,642	0.082	2,178,555	Comparator	Comparator	No	No	n.a
52	Noyes et al. (2011) [52]	20,6048	0.126	1,635,299	187,401	0.126	1,487,306	Comparator	Comparator	No	No	n.a
53	Nuijten and Hutton (2002) [53]	449,156	3.3	136,108	321,308	3.3	97,366	Comparator	Comparator	No	No	n.a
54	Nuijten and Hutton (2002) [53]	170,222	3.3	51,582	150,616	3.3	45,641	Comparator	Comparator	No	No	n.a
55	Pan et al. (2012) [54]	113,784	5.1	22,311	175,610	5.1	34,433	Assessed intervention	Assessed intervention	No	No	$50,000
56	Pan et al. (2012) [54]	36,789	1.9	19,363	86,223	1.9	45,381	Assessed intervention	Assessed intervention	No	No	$50,000
57	Soini et al. (2017) [55]	16,077	0.477	33,704	2,000	0.477	4,193	Assessed intervention	Assessed intervention	No	No	€68,000 €
58	Soini et al. (2017) [55]	9,346	0.388	24,088	− 2,000	0.388	− 5,155	Assessed intervention	Assessed intervention	Yes, the assessed intervention becomes dominant (not only cost-effective)	No	€68,000
59	Soini et al. (2017) [55]	15,216	0.264	57,636	8,000	0.264	30,303	Assessed intervention	Assessed intervention	No	No	€68,000
60	Soini et al. (2017) [55]	35,876	0.144	249,139	33,000	0.144	229,167	Comparator	Comparator	No	No	€68,000
61	Soini et al. (2017) [55]	30,405	0.125	243,240	27,000	0.125	216,000	Comparator	Comparator	No	No	€68,000
62	Soini et al. (2017) [55]	75,362	− 0.268	− 281,201	85,000	− 0.268	− 317,164	Comparator	Comparator	No	No	€68,000
63	Taheri et al. (2019) [56]	− 3,162	0.56	− 5,646	− 3,723	0.63	− 5,910	Assessed intervention	Assessed intervention	No	No	n.a
64	Tosh et al. (2014) [57]	466	0.046	10,137	1,144	0.046	24,897	Assessed intervention	Assessed intervention	No	No	£20,000–30,000
65	Tosh et al. (2014) [57]	466	0.024	19,783	1,144	0.024	47,667	Assessed intervention	Comparator	Yes	Yes, the assessed intervention is no longer cost-effective and will not be adopted	£20,000–30,000
66	Touchette et al. (2003) [58]	7,047	0.121	58,240	− 5,056	0.121	− 41,785	Comparator	Assessed intervention	Yes, when social costs are included, the incremental costs changed from positive to negative	Yes, the assessed intervention switched from non-cost-effective to dominant and, hence, will be adopted	$50,000
67	Touchette et al. (2003) [58]	69,502	0.2052	338,704	50,412	0.2052	245,673	Comparator	Comparator	No	No	$50,000

A change in conclusions was identified when, in cases where social costs were included, the conclusion about the adoption of a new technology was modified (i.e., from the healthcare perspective, the ICUR was above the threshold value and, hence, the assessed technology will not be implemented as it is not cost-effective. However, when social costs were included, the ICUR was modified so that it lies below the corresponding threshold and the technology should be implemented). A change in the results implied that the ICUR from a societal perspective might have changed the recommendation made by authors, but it could also mean a significant change in the ICUR (e.g., switching the intervention to dominant, when from a healthcare perspective it was not)

The inclusion of social costs changed the incremental costs of the assessed intervention versus its comparator in 15 economic evaluation estimations. Although still positive (having higher costs than the comparator) when social costs were considered, the incremental costs were reduced so as to make the corresponding ICUR fall below the comparative threshold in three cases. In another one, the opposite pattern was observed. Moreover, in eight additional estimations, from the societal perspective the incremental costs of the assessed intervention changed from being positive to negative, implying that the evaluated intervention was cost-saving against its comparator. Three additional estimations showed the contrary trend: positive incremental costs from the societal perspective, but cost-saving from the perspective of the healthcare payer/provider.

In view of the aforementioned changes in incremental costs due to the inclusion of social costs (informal care costs and/or productivity losses), the conclusions were modified in ten EEs (almost 15% of the EEs analysed in the review). Economic evaluations 35, 36 and 37 showed that, when informal care and labour productivity losses were included, the procedure that was the subject of analysis became cost-effective, as its cost per additional QALY lay below the €30,000 threshold set by the authors, compared to its lack of cost-effectiveness from the healthcare payer’s perspective. Moreover, in three EEs, the inclusion of social costs (labour productivity losses only in the case of EEs 14 and 15 and both types of social costs for EE 66) resulted in a more dramatic change: the assessed interventions became dominant from the societal perspective (lower costs and higher health gains), when, from the healthcare payer’s perspective, they were not cost-effective. Conversely, in EEs numbers 9, 10 and 12, the assessed intervention was cost-effective when the analysis was performed from the healthcare payer’s perspective, but it became dominated by the comparator when social costs were included, owing to higher incremental costs. Economic evaluation 65 showed that once labour productivity losses were considered, the intervention was no longer cost-effective, as it was from the perspective of the healthcare payer/provider.

Moreover, and although not involving any change in the conclusions as in the ten estimations described above, the inclusion of social costs did show a change in results in five additional EEs (more than 7% of the total number of EEs): the EEs numbered 17, 20, 33, 45 and 58 became not only cost-effective, as they were already from the healthcare payer/provider’s perspective, but also dominant after the inclusion of social costs, because, in addition to being better in terms of health outcomes, they were cost- saving.

From the healthcare payer/provider’s perspective, 12 EEs (17.91%) reported negative incremental costs (estimations number 6, 7, 8, 9, 10, 12, 16, 21, 22, 46, 47 and 63), pointing towards the assessed intervention being cost-saving against the comparator. However, only eight (11.94%) of them led to the conclusion that the intervention was dominant (estimations number 6, 7, 16, 21, 22, 46, 47 and 63). On the other hand, if the societal perspective was applied, 17 estimations (25.37%) showed negative incremental costs (estimations number 6, 7, 8, 14, 15, 16, 17, 20, 21, 22, 33, 45, 46, 47, 58, 63 and 66), all of which, apart from 1 EE, proved to be dominant, leading to lower costs and higher gains in QALY. Another remarkable result is the one obtained in the two EEs (number 21 and 22) from the study carried out by Hettle et al. (2018) [40] and in estimation number 63 by Taheri et al. (2019) [56] when comparing the results from the healthcare payer’s perspective with those from the societal perspective. In all the scenarios, the assessed intervention was cost-saving and led to gains in health, but both economic and QALY outcomes were higher from the societal perspective, when carers’ utilities were incorporated, showing that pharmaceutical interventions also reported benefits to informal caregivers. These were the only studies that additionally took into account the informal carers’ utilities when applying the societal perspective.

With regard to the incremental cost-utility ratios (ICURs), from the healthcare perspective, 18 (26.87%) EEs (number 8, 9, 10, 12, 17, 18, 19, 20, 33, 44, 45, 55, 56, 57, 58, 59, 64 and 65) proved to be cost-effective (ICUR below the corresponding threshold value). From the societal perspective, 12 ICURs were below the threshold and were thus cost-effective (EEs number 8, 18, 19, 35, 36, 37, 44, 55, 56, 57, 59 and 64).

**Fig. 2:**
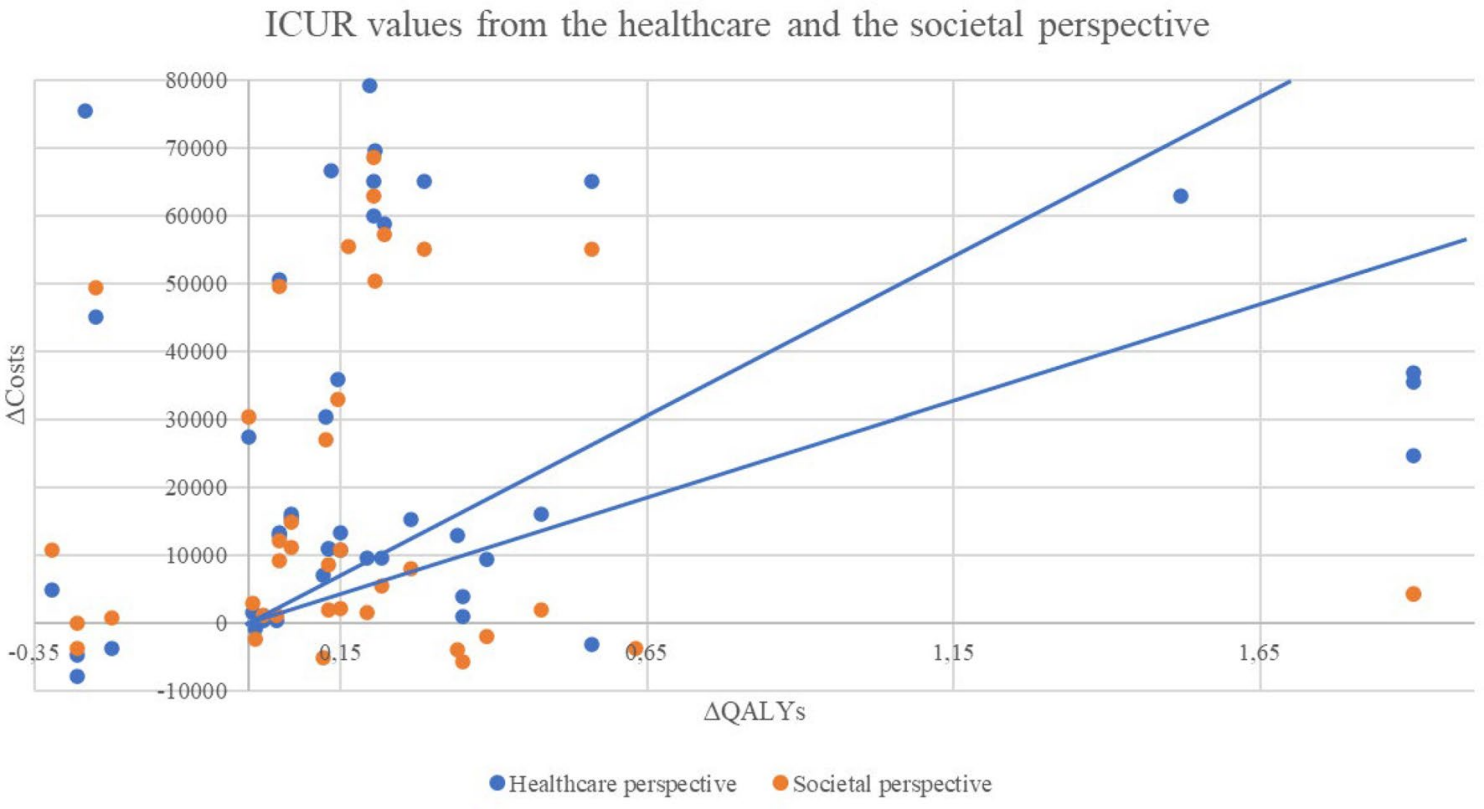
Incremental cost-utility ratios from the healthcare and societal perspectives

Figure 2 shows the dispersion of the costs and QALYs of the 67 economic evaluation estimations included, according to the perspective applied. The most noticeable effect is that several interventions that had intermediate values of between €30,000 and 50,000/QALY fall below the threshold of €30,000/QALY or are even cost-saving when the societal perspective is applied.

## Discussion

In this review, we sought to explore changes that might occur due to the application of different costing perspectives (that of the health financier/provider or that of society) to economic evaluations of MS interventions. To fill the gap in the existing literature, the objective of this review was twofold. First, to identify the number of economic evaluations, carried out from a societal perspective, of the treatments related to multiple sclerosis. Second, to investigate the effect that the choice of perspective (societal versus healthcare provider’s or payer’s perspective) has on the results and conclusions of the economic evaluations implemented in this area. Our analysis shows that the results and possible recommendations for decision-makers can differ depending on the perspective selected.

In relation to the first objective, the proportion of articles about economic evaluations that include the societal perspective in the field of multiple sclerosis is noteworthy. Almost half of the articles (47%; 42 out of 90 papers) used the societal perspective. This proportion is notably higher than that found in the area of treatments for rare diseases, where a review found that only 11% of the studies included a societal perspective[59], a ratio slightly higher than that found for the area of depression (42%) [60], but lower than that found for interventions in Alzheimer’s disease (58%) [61].

In the economic evaluations which include a societal perspective, a further difference was found between the types of social costs that were included in the estimations for MS and those included in the economic evaluations of other diseases. In the case of MS, the majority of EEs (about 90%) that considered the societal perspective analysed costs associated with labour productivity losses. This finding is quite similar to that for diseases such as depression (95% of EEs that considered the societal perspective estimated labour productivity losses) and rare diseases (about 80%). However, in other diseases such as Alzheimer’s, this figure barely reaches 3% [59–61]. The differences are even higher when considering the existence of informal care costs. In multiple sclerosis, about 65% of the EEs that considered the societal perspective included informal care costs, only surpassed in studies of Alzheimer’s, 97% of which included these costs, whereas for diseases such as depression and rare diseases, the opposite trend was shown, with only 29 and 22% of the respective studies including informal care costs.

Finding the reason for these differences in the weight and/or the presence of informal care costs and productivity losses, depending on the diseases considered, is neither clear nor intuitive. They could be explained by the nature of the disease (for example, by the age at onset). Thus, in the case of Alzheimer’s, rare diseases or multiple sclerosis, the costs may be shared between the patients (through productivity losses) and the family (through informal care costs), whereas in the case of depression, the burden could be mainly supported by individuals (through loss of work). In case of the latter disease, it would seem that the burden is still usually considered as a problem for the patient as an individual (through loss of work), and the financial strains on the affected family are not taken into consideration. As has been previously stated, multiple sclerosis is often diagnosed at early ages, even during childhood or early adulthood [11], and leads to disability and a reduced health-related quality of life over time [16, 17]. As a result, societal costs, such as productivity losses and formal and informal long-term care costs, can be incurred from early ages and throughout the rest of life [13]. On the other hand, the differences might be explained by the fact that the inclusion of certain types of costs, such as informal care costs, has not been considered until recently in the literature about some diseases, and it is still a challenge to be faced in cost-of-illness and economic evaluation studies [62, 63].

It is not easy to know why the societal perspective is more evident in connection with some diseases compared to others since there is evidence that the social costs associated with them are very substantial. In the case of MS, two studies conducted as long as 20 years ago highlighted the importance of informal care costs in some European countries [64], especially in the United Kingdom [46, 64] and in Italy [65], where they were the main societal costs, and this was confirmed by later European studies[23, 66]. In fact, social costs can be as high as healthcare costs in the four diseases previously mentioned (Alzheimer’s disease, rare diseases, depression and MS). In any case, it is surprising that the proportion of economic evaluations that consider the societal perspective was not higher, when a large number of countries, such as Sweden, the Netherlands and France [67–69], either recommend using the societal perspective or point out the importance of using both the societal perspective and that of the healthcare financier, as in Spain and Italy [70, 71]. Other countries have opted for the perspective of the healthcare funder but allow, as a supplementary analysis, the inclusion of the societal perspective (Australia, Canada, the Baltic countries, Belgium and Poland, among others) [72–76]. Even in the case of England and Wales, although the main perspective is that of the healthcare funder, in appropriate cases the inclusion of personal social services (PSS) is allowed [77].

With regard to the second objective of the review, the inclusion of social costs modified the incremental costs of the assessed interventions versus their comparators in 15 economic evaluation estimations. In 10 of them (14.9% of the 67 economic evaluations reviewed in this work), the use of the societal perspective could modify the recommendations/conclusions of the evaluations. In six cases, the consideration of social costs made the evaluated intervention cost-effective compared to its comparator, but in four other cases the effect was the opposite, and the evaluated intervention was no longer cost-effective against its comparator. Even though the inclusion of social costs did not affect the adoption of the assessed intervention in all the estimations, it is also worth mentioning that it did produce a change in results in 7.5% of the economic evaluations analysed, which changed from having a good cost-effectiveness ratio from a healthcare perspective to being dominant from a societal point of view. When comparing these results with other diseases (such as depression, Alzheimer’s and rare diseases), it was observed that, even though consideration of the societal perspective had a positive influence by changing the interventions in those diseases to dominant (cost-saving) [59–61], this positive effect was weaker than in the case of MS, in which the inclusion of social costs led to a larger number of changes in the incremental costs. This might be explained by the nature of the interventions performed within these diseases, as in the case of MS they are mainly pharmaceutical interventions with a lifetime horizon. In addition, when analysing the changed conclusions in some interventions, some reasons could be considered. First, because when the interventions are medical procedures, the differences in social costs are usually smaller than those in non-medical procedures, such as pharmaceutical interventions [61]. Second, because for those interventions whose time horizon is longer (10 years, even more, or even lifetime), the difference in social costs is greater than for those interventions whose time horizon is shorter (less than 3 years).

From a methodological point of view, the lack of transparency in many of the studies analysed is particularly worrying. In two-thirds of the articles which included the costs of informal care as part of the costs analysed from the societal perspective, the method of assessing informal care time was not made explicit by the original authors of the study. Of the six articles which specified the method used, three used the opportunity-cost approach and in the other three, the replacement-cost approach was used. Of the 26 articles that included productivity losses, 10 of them (38.5%) did not specify the method used. Of the 16 studies that indicated the approach, 15 used the human capital as the method, while only one study used the alternative approach of friction costs. It should be noted that high heterogeneity was identified in the items valued as productivity losses. Some articles only included absenteeism, while other papers included presenteeism, permanent sick leave, early retirement, and premature mortality.

Some limitations should also be taken into account when interpreting our findings. However, most of these limitations were due to the lack of homogeneity in the information provided by the original authors. First, since no homogeneous methodology was observed among the studies included (i.e., method used to value productivity losses and/or informal care costs, detailed information about social cost components), the comparability between studies might be compromised. Second, we did not aim to reassess the evaluated healthcare interventions in each original study, but to review whether the inclusion of the societal perspective could modify the conclusion. However, the authors considered different time horizons, discount rates, healthcare costs and cost-effectiveness thresholds, which may also limit the comparability of results. Moreover, the stage of the disease might be a relevant factor behind the economic burden of the condition, but there is no consistency in relation to this question, since 10 studies did not specify the degree of severity [33, 36, 39, 42, 44, 48, 51–54, 57], while 11 studies referred to relapsing–remitting MS individually [31, 34, 35, 37, 38, 40, 41, 43, 50, 55, 56] and the other 8 studies considered slow or secondary-progressive MS, solely or jointly with relapsing–remitting MS. Lastly, the search strategy in relation to the databases used might be subject to debate. However, we complemented our search launched in Medline by additionally using the CEA Registry of Tufts University, which applies an algorithm also launched in Medline and a systematic review process [27, 78].

To conclude, the systematic review performed indicates that the adoption of a societal perspective would modify the results of economic evaluations of MS-related interventions, as well as the conclusions about their implementation. Therefore, consideration of the perspective used in the economic evaluations carried out in the field of MS, far from being neutral, can lead to important consequences in relation to the information generated for decision-makers. Excluding the societal perspective when performing economic evaluations in diseases such as MS could lead to the omission of relevant information for decision-makers, and could even result in a misguided recommendation about whether a new and available treatment should be adopted or not. In addition, to be truly social, the societal perspective should also include the effect on caregivers’ health status. It is remarkable that, although almost two-thirds of the selected economic evaluations included informal care costs, only two studies [40, 56] also considered the effects of the assessed intervention on the caregivers’ health. Such effects could be highly important in view of the economic burden borne by caregivers of people with MS, and because interventions aimed at improving the care of people with this disease have been shown not only to lead to better states of health of both caregivers and care receivers [79, 80], but also to maintain those positive effects even after the intervention has finished [80], suggesting that caregivers might be an appropriate and independent target for more focussed MS-related therapeutic strategies. In fact, one of the major recommendations of recent caregiver reports [81–83] is to include the caregiver in the care receiver’s care plan. Hence, the effect of implementing healthcare interventions on the well-being and health of those providing care, which might additionally entail the identification of consequential effects, may come to prominence as a need for methodological improvement that should be taken into account in future studies. Moreover, in future studies, the stage of MS should also be a key factor when assessing the economic evaluation of new drugs, because some studies have already provided evidences of the effectiveness of pharmacological treatments in delaying the progression to secondary-progressive MS and evidence of the effectiveness of an early start of the treatment [84, 85]. In addition, when interpreting the results by geographical location, the distribution of total costs between healthcare costs, professional and non-professional care costs, and labour productivity losses might be subject to country-specific employment and social policies, as there are notable differences between countries [86, 87]. Future analyses could aim to clarify the way in which the composition of costs differs among the countries where the economic evaluations are performed, as well as their relationship with social protection policies.

For ease of comparison, results are shown in additional euros per additional QALY. For this, the euro-currency exchange rates of the year of each article were applied. The values were not updated to any base year since the efficiency thresholds applied as a usual reference are usually kept constant over several years. In this sense, and to facilitate the interpretation of the results, two vectors were drawn with the values of €30,000/QALY and €50,000/QALY. These values were adopted as they are frequently cited thresholds in the economic evaluation literature.
